# Bedside Multimodal Ultrasonography for the Detection of Intracranial Hypertension in Severe Adolescent Preeclampsia: A Case Report and Screening Proposal

**DOI:** 10.7759/cureus.105164

**Published:** 2026-03-13

**Authors:** Sandra Olaya, Sergio David Angulo, Isabella Montealegre, Juan Diego Trujillo Loaiza, Natalia Buelvas, Juan Pablo Franco, Héctor Fabio Castaño

**Affiliations:** 1 Gynecology, Caja de Compensación Familiar de Risaralda, Salud Comfamiliar, Pereira, COL; 2 Grupo de Investigación Biomedicina, Faculty of Medicine, Fundación Universitaria Autónoma de las AméricasInstitución Universitaria Visión de las Américas, Pereira, COL; 3 Teaching and Research Unit (UID), Caja de Compensación Familiar de Risaralda, Salud Comfamiliar, Pereira, COL; 4 General Surgery, Universidad de Manizales, Manizales, COL; 5 Anesthesiology, Universidad de Manizales, Manizales, COL; 6 General Practice, Universidad de Manizales, Manizales, COL; 7 Anesthesia and Critical Care, Hospital Universitario San Jorge, Pereira, COL

**Keywords:** eclampsia, intracranial hypertension, ocular ultrasonography, optic nerve sheath diameter, preeclampsia, transcranial doppler

## Abstract

Preeclampsia is a leading cause of maternal morbidity and mortality worldwide and is frequently complicated by cerebral edema and elevated intracranial pressure (ICP). Noninvasive neuromonitoring tools such as transcranial Doppler (TCD) and ocular ultrasonography may help detect intracranial hypertension before overt neurological deterioration. We report a 17-year-old primigravida at 34 weeks of gestation who presented with severe preeclampsia and a hypertensive crisis, but without focal neurological deficits. Laboratory tests confirmed severe preeclampsia, and an emergency caesarean section was performed with a good neonatal outcome. Postoperatively, bedside TCD of the middle cerebral artery showed an elevated pulsatility index (PI = 1.25) and resistance index (RI = 0.68). At the same time, ocular ultrasound demonstrated an enlarged optic nerve sheath diameter and optic disc protrusion, both consistent with increased ICP despite the patient's normal neurological exam. This case illustrates the potential role of combined TCD and ocular ultrasonography for early, noninvasive assessment of intracranial hypertension in severe preeclampsia, particularly in adolescent patients. We propose a two-step bedside ultrasound protocol to identify subclinically elevated intracranial pressure.

## Introduction

Preeclampsia is a multisystem pregnancy disorder characterized by variable degrees of placental malperfusion with the release of soluble factors into the circulation [1]. These factors cause maternal vascular endothelial injury, which leads to hypertension and multi-organ complications such as pulmonary edema, kidney injury, increased liver enzymes, and neurological complications [1-2]. Preeclamptic encephalopathy is a term used to describe the spectrum of neurologic symptoms, including headache, visual disturbances, and seizures, with reversible subcortical vasogenic cerebral edema [3]. This constellation of symptoms is referred to as reversible posterior leukoencephalopathy syndrome (RPLS), also called posterior reversible encephalopathy syndrome (PRES). The risk of impairing long-term maternal neurocognitive function is nine times higher in these patients compared to those with normotensive pregnancies. This deficit remains statistically significant up to 19 years postpartum [4].

Magnetic resonance imaging (MRI) reveals cerebral edema in 71% to 100% of patients with severe preeclampsia [5-7]. Cerebral edema in preeclampsia is predominantly vasogenic, driven by endothelial leakage that disrupts pressure homeostasis and ultimately elevates intracranial pressure (ICP) [8]. To monitor these changes at the bedside, clinicians use tools like optic nerve sheath diameter (ONSD) and transcranial Doppler (TCD). ONSD measures the thickness of the membrane surrounding the optic nerve, which expands when brain pressure rises, acting as an indirect "gauge" of ICP. TCD uses ultrasound to assess the speed and resistance of blood flow within the brain's arteries.

A non-invasive bedside method for recognizing cerebral edema and increased ICP could, therefore, be beneficial in the management of patients with severe preeclampsia. Neuromonitoring tools, such as TCD ultrasonography, are gaining critical relevance [9]. In severe preeclampsia, a low pulsatility index (PI) and resistance index (RI) in the middle cerebral artery (MCA) indicate compensatory vasodilation that often precedes neurological symptoms [10]. However, as ICP continues to rise and exceeds the brain's compensatory capacity, the PI may subsequently increase, reflecting high arterial inflow resistance. Conversely, although a PI >1.1 is generally associated with elevated ICP in adult populations, its diagnostic validity in pregnant patients remains unclear. Similarly, ocular ultrasound of an enlarged ONSD has been associated with elevated ICP and papilledema in females with severe preeclampsia [11]. The progression from preeclampsia to severe neurological compromise is influenced by several risk factors, including chronic hypertension, nulliparity, pregestational diabetes, and obesity [12]. However, a significant gap remains: no standardized measurement or bedside protocol has been validated in adolescents with preeclampsia, where physiological responses may differ from those of adults.

## Case presentation

Initial assessment and diagnosis

A 17-year-old primigravida at 34 weeks of gestation was admitted to a tertiary-level hospital presenting with a one-week history of peripheral edema, photopsia, tinnitus, diffuse throbbing headache, and epigastric pain. Her obstetrical history was significant for recurrent urinary tract infections during the current pregnancy, requiring treatment with cephalexin. She had attended four prenatal visits, during which her blood pressure remained within normal limits (average 110/70 mmHg). However, an obstetric ultrasound performed at 32.4 weeks revealed an elevated pulsatility index (PI) in the uterine arteries with protodiastolic notching, indicating a risk of intrauterine growth restriction (IUGR). Based on these Doppler findings, alpha-methyldopa was initiated. Her medical history was otherwise unremarkable.

The patient was transferred to the Maternal Intensive Care Unit (MICU). Initial examination showed a hypertensive crisis (170/100 mmHg) that required arterial line placement. The neurological examination revealed no focal motor or sensory deficits. The patient was evaluated for evidence of end-organ dysfunction; results are shown in Table 1. 

**Table 1:** Laboratory data at admission. AST: aspartate aminotransferase, ALT: alanine aminotransferase, LDH: lactate dehydrogenase, BE: excess base. Arterial blood gases were taken at room air.

Parameter	Patient	Reference range
pH	7.42	7.35-7.45
PCO₂ (mmHg)	28	35-45
PO₂ (mmHg)	102	80-100
HCO₃ (mmol/L)	20.4	22-29
BE (mmol/L)	-6.9	-2 to +2
Sodium (mEq/L)	133	135-145
Potassium (mEq/L)	3.60	3.5-5.0
Calcium (mmol/L)	1.14	1.1-1.4
Lactate (mmol/L)	2.90	<2.00
LDH (U/L)	158	<280
ALT (U/L)	17	7-56
AST (U/L)	22	8-48
Leukocytes (cells/μL)	9320	4000-11000
Neutrophils (cells/μL)	7270	1500-8000
Hemoglobin (g/dL)	9.40	>10.10
Hematocrit (%)	29.1	30-55
Platelets (cells/μL)	237000	150000-350000
Creatinine (mg/dL)	0.6	0.4-1.1
Proteinuria (g/24h)	1.045	<0.15

Although laboratory markers were within normal limits, the patient was diagnosed with severe preeclampsia based on the persistent hypertensive crisis and the presence of severe neurological symptoms (diffuse throbbing headache and tinnitus); seizure prophylaxis with magnesium sulfate infusion, antihypertensive therapy with nifedipine, and antenatal corticosteroids were initiated. Initial laboratories revealed maternal anemia; one unit of packed red blood cells (PRBCs) was transfused before delivery.

Delivery and immediate postoperative

Following the completion of antenatal corticosteroids, the gynecologist performed an emergency cesarean section. The procedure required the transfusion of two additional units of PRBCs. A neonate was delivered weighing 1,790 g and measuring 40 cm in length, with Apgar scores of 7 at 1 and 5 minutes. The infant was transferred to the Neonatal Intensive Care Unit (NICU). Postoperatively, the mother was transferred back to the MICU. Blood pressure (140/100 mmHg) required the addition of a second-line antihypertensive agent (nicardipine). Physical examination showed no neurological deficit at that time.

Bedside neuromonitoring with TCD and ocular ultrasound

**Figure 1:**
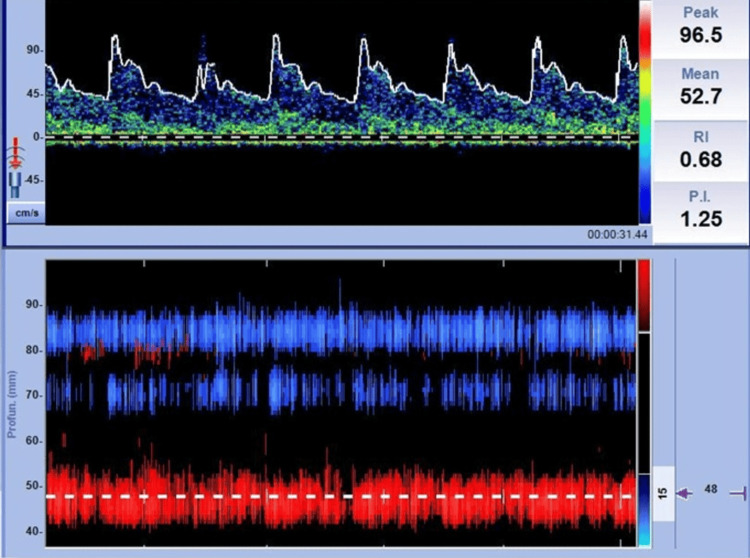
TCD of the MCA showing a PI of 1.25 and an RI of 0.68. TCD: Transcranial Doppler; MCA: Middle Cerebral Artery; PI: Pulsatility index; RI: Resistance index, Source: original work

On the first postpartum day, the medical team performed a TCD with a 2-MHz transducer. The transtemporal window was utilized to insonate the proximal segment of the MCA at a depth of 30-60 mm. The waveform demonstrated a PI > 1, indicating elevated resistance in the MCA (Figure 1).

**Figure 2:**
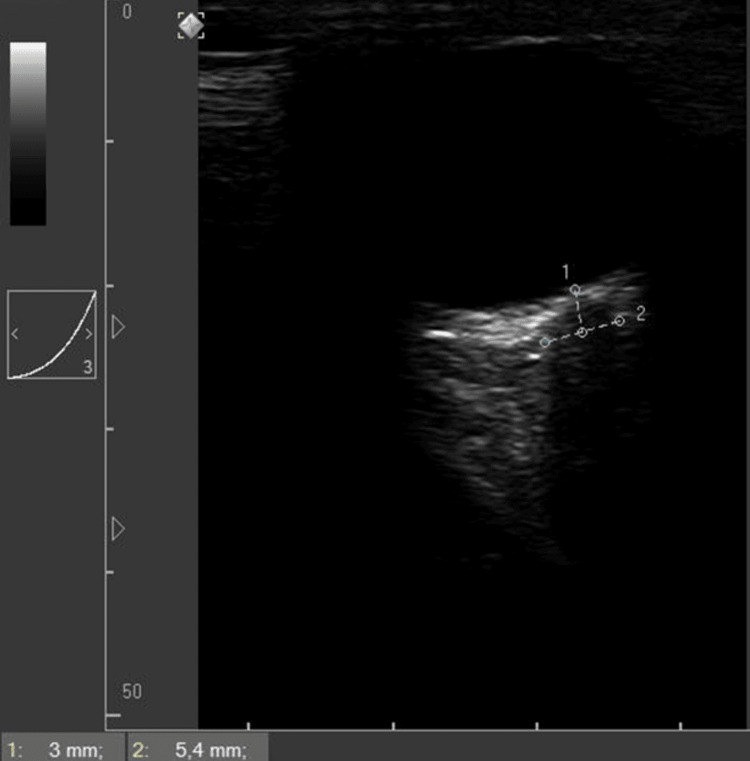
Ocular ultrasonography. (1) Optic disc height (ODH): 3.0 mm (Reference: < 0.5 mm), showing significant protrusion indicative of papilledema. (2) Optic nerve sheath diameter (ONSD): 5.4 mm (Reference: 4.6–5.0 mm), reflecting increased intracranial pressure. Measurements were obtained 3 mm posterior to the globe. Blood pressure at measurement: 125/80 (95)

Ocular ultrasonography was performed using a 7.5 MHz linear transducer applied to the closed upper eyelid to visualize the optic nerve sheath. The ONSD was measured 3 mm posterior to the globe, revealing a thickness >5 mm (Figure 2). 

Additionally, optic nerve head protrusion was noted, suggestive of elevated intracranial pressure despite the patient being clinically asymptomatic. Upon completion of the 24-hour magnesium sulfate infusion and hemodynamic stabilization, the patient was transferred to the general ward and discharged the following day with outpatient follow-up. In Table 2, we summarize the timeline since admission.

**Table 2:** Timeline. BP: Blood pressure; MAP: Mean arterial pressure; ONSD: Optic nerve sheath diameter; ODH: Optic disc height; TCD: Transcranial Doppler

Time Point	Clinical Status	Glasgow Coma Scale	BP/MAP (mmHg)	Medical Management	ONSD (Right)	ODH (Right)	TCD (PI)
Admission	Diffuse headache, tinnitus, and hypertension	15	170/110 (130)	MgSO₄ bolus, alpha-methyldopa	Not done	Not done	0.8
Post-Delivery (6h)	Hypertension	15	140/100 (113)	MgSO₄ infusion, alpha-methyldopa, nifedipine	Not done	Not done	Not done
Day 3 (Discharge)	Asymptomatic	15	125/80 (95)	Nifedipine	5.4 mm	3.0 mm	1.25

## Discussion

From a pathophysiological perspective, severe preeclampsia is associated with alterations in cerebral autoregulation and the integrity of the blood-brain barrier in the context of systemic endothelial dysfunction and angiogenic imbalance [12]. This environment favors extravasation of fluid into the cerebral parenchyma and the development of vasogenic edema. This phenomenon can occur even in the absence of overt neurological manifestations, which helps explain, at least in part, the burden of neurological complications associated with hypertensive disorders of pregnancy [13]. The resulting increase in intracranial pressure is transmitted to the peripapillary subarachnoid space, distending the optic nerve sheath, which supports the use of ONSD as an indirect surrogate for intracranial pressure [14].

Based on this, ultrasonographic measurement of ONSD is proposed as an initial step in non-invasive neurological assessment for risk stratification. Several observational studies have consistently shown higher ONSD values in patients with severe preeclampsia compared to normotensive pregnant women, both during pregnancy and in the immediate postpartum period [15]. The pilot cohort study by Dubost et al. reported that approximately one-fifth of patients with preeclampsia had elevated ICP values (ONSD > 5.8 mm), which tended to normalize in the postpartum period [16]. A finding later replicated in cohorts with a higher proportion of patients meeting criteria for severity and neurological manifestations [17].

The findings in the present case are consistent with this evidence and reinforce the utility of ONSD in identifying patients at higher risk of neurological compromise, even when clinically asymptomatic. This aspect is particularly relevant in critical obstetrics, where symptoms such as headaches, visual disturbances, or changes in mental status may be subtle or fluctuating and where decision-making often occurs amid clinical uncertainty.

ONSD should be interpreted as a screening tool rather than a direct measurement of intracranial pressure. Meta-analyses and descriptive studies have demonstrated a moderate correlation between ONSD and intracranial pressure, measured invasively or estimated from neuroimaging, in non-obstetric populations. However, there is significant heterogeneity in measurement techniques and proposed cutoff points [18]. In obstetric populations, evidence remains limited and predominantly observational, necessitating cautious interpretation of absolute values and their clinical relevance.

**Figure 3:**
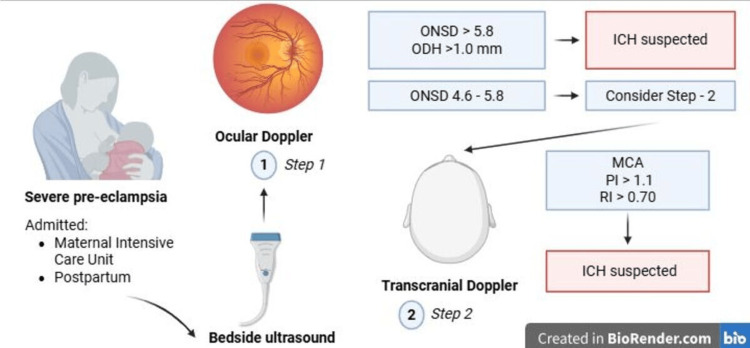
Algorithm: two-step bedside screening for elevated ICP in severe preeclampsia. ICH: Intracranial hypertension, ICP: Intracranial pressure, ONSD: Optic nerve sheath diameter, ODH: Optic disc height, MCA: Middle cerebral artery, PI: Pulsatility index, RI: Resistance index. Source: Created in https://BioRender.com.

In this context, it is pertinent to discuss the reported cut-off points in the literature. Various studies have suggested ONSD thresholds ranging from 5.0 to 5.8 mm to indicate a higher likelihood of intracranial involvement, with variations attributable to population, methodological, and clinical differences [18]. We propose a conceptual approach (Figure 3) in which ONSD, ODH, and TCD measurements are represented as a continuum rather than dichotomous variables, underscoring their role as risk-stratification tools rather than definitive diagnostic criteria. These findings represent risk markers rather than definitive diagnostic confirmation of elevated ICP.

Alongside the structural evaluation provided by ONSD, TCD and ultrasound techniques have been described as useful bedside tools for exploring cerebral hemodynamics and autoregulation in patients with preeclampsia and eclampsia, as well as in other critical maternal conditions [19]. In the available literature, its use has been primarily descriptive and aimed at understanding the pathophysiology of hypertensive disorders of pregnancy. In this framework, TCD and ONSD measurements should be understood as complementary approaches targeting different domains.

Within this conceptual framework, the value of ONSD lies in its technical simplicity, reproducibility, and low training requirements, characteristics that enhance its applicability in obstetric intensive care units and resource-limited settings. These strengths must be considered alongside its inherent limitations: indirect measurement, susceptibility to inter-observer variability, and cut-off points not uniformly validated in obstetric populations [20].

These considerations support the need for prospective and multicenter studies that integrate bedside structural and hemodynamic tools, which will allow a more precise definition of the role of ONSD in neurological risk stratification and its potential impact on maternal and neonatal outcomes, particularly in populations with a high burden of hypertensive disorders of pregnancy.

This case demonstrates the feasibility of combining TCD and ocular ultrasound to detect subclinical ICH in neurologically normal patients with severe preeclampsia. Limitations include single measurements without invasive ICP correlation or long-term follow-up. Prospective validation of the proposed algorithm is warranted, particularly in adolescent and resource-limited settings.

## Conclusions

The clinical significance of cerebral hemodynamic changes in severe pre-eclampsia remains an active area of research. Clarifying the predictive, diagnostic, and prognostic value of these biomarkers is essential for optimizing the management of elevated ICP in preeclampsia. Furthermore, mitigating maternal cerebral hemodynamic compromise could be key to preventing long-term complications. In conclusion, this case demonstrates that non-invasive neuromonitoring tools are valuable for identifying sonographic markers suggestive of increased ICP in adolescent patients with severe preeclampsia.
